# Case Report: subhepatic appendicitis complicated by hepatic abscess as the initial presentation of primary diffuse large B-cell lymphoma of the appendix: first reported case

**DOI:** 10.3389/fonc.2025.1591905

**Published:** 2025-06-16

**Authors:** Hao Chen, Miao Yu, Zhanping Chang, Yang Li, Lei Fan, Dake Shang, Huili Zhang

**Affiliations:** ^1^ General Surgery, China Aerospace Science & Industry Corporation 731 Hospital, Beijing, China; ^2^ Pathology Department, China Aerospace Science & Industry Corporation 731 Hospital, Beijing, China

**Keywords:** subhepatic appendicitis, liver abscess, diffuse large B-cell lymphoma, non-Hodgkin lymphoma, appendectomy, case report

## Abstract

Subhepatic appendicitis complicated by hepatic abscess is an uncommon clinical condition due to anatomical variations in the appendix’s position. Here, we report an unusual case of a 56-year-old male who initially presented with right abdominal pain and fever, misdiagnosed as bilateral kidney stones and urinary tract infection. Subsequent abdominal CT(Computed Tomography) scans revealed subhepatic appendicitis with a hepatic abscess located in segment VI of the liver. The patient underwent successful laparoscopic appendectomy and hepatic abscess drainage. Unexpectedly, histopathological examination of the appendix demonstrated diffuse large B-cell lymphoma (DLBCL), a rare form of primary appendiceal non-Hodgkin lymphoma. Immunohistochemistry confirmed the diagnosis, with tumor cells positive for CD20, CD79a, Bcl-6, and c-Myc, and a high Ki-67 proliferative index (>90%). This case highlights the diagnostic challenges associated with atypically positioned appendicitis and underscores the importance of pathological evaluation in detecting rare underlying malignancies.

## Introduction

Subhepatic appendicitis is an uncommon clinical entity characterized by an anatomically atypical location of the appendix beneath the liver, occurring in approximately 0.08% of acute appendicitis cases (1). This rare anatomical variation is typically associated with embryological anomalies such as intestinal malrotation or incomplete descent of the cecum (2). The abnormal location predisposes patients to atypical clinical presentations, often mimicking hepatobiliary or renal diseases, which can delay diagnosis and treatment (1). Moreover, complications such as hepatic abscess formation further complicate clinical management. Primary diffuse large B-cell lymphoma (DLBCL) involving the appendix is exceedingly rare and poses additional diagnostic challenges due to nonspecific clinical manifestations (6). Here, we present the first reported case of subhepatic appendicitis complicated by hepatic abscess as the initial clinical manifestation of primary diffuse large B-cell lymphoma of the appendix, highlighting the importance of awareness and comprehensive pathological evaluation for accurate diagnosis and optimal patient outcomes.

## Case presentation

**Figure 1:**
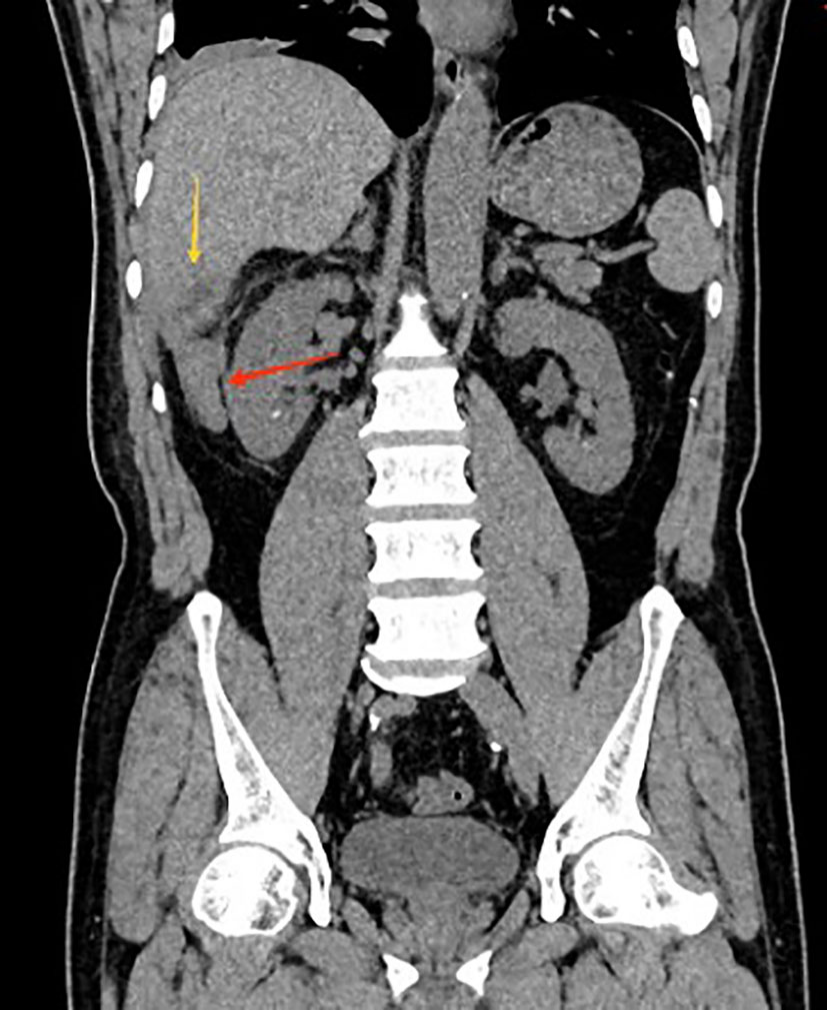
Coronal CT showing subhepatic appendicitis (red arrow) with associated hepatic abscess in segment VI (yellow arrow).

**Figure 2:**
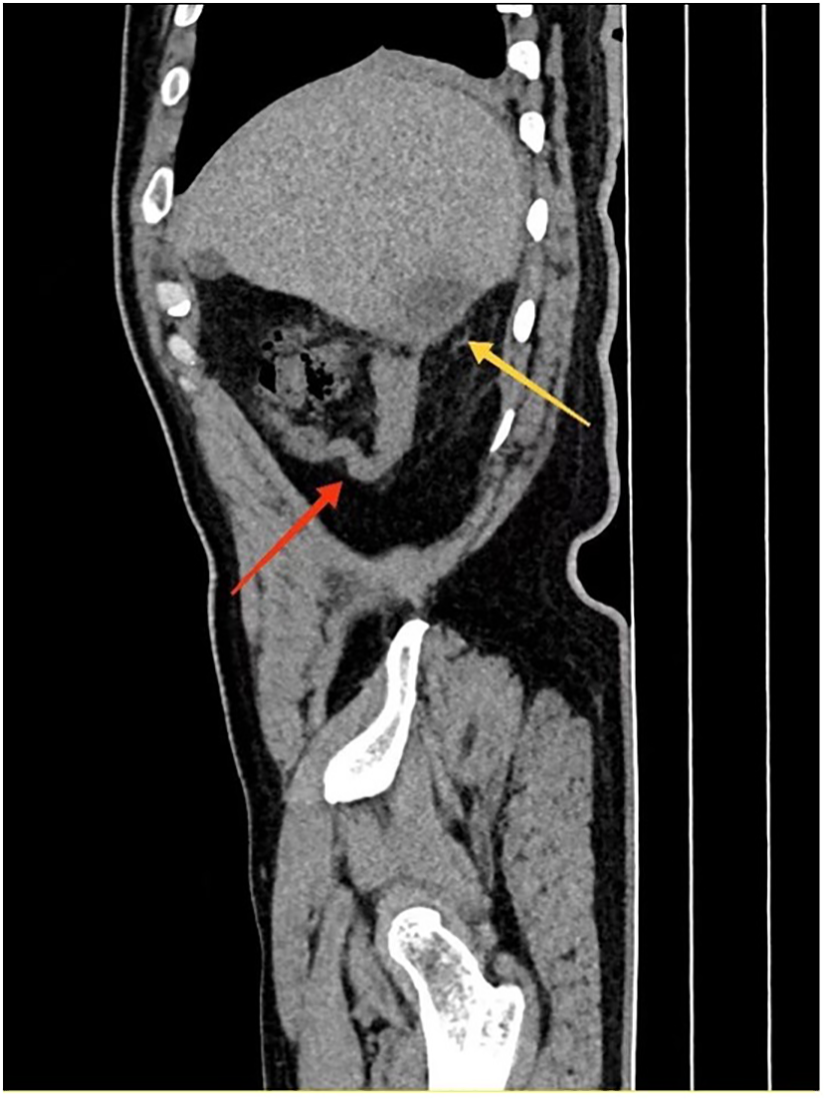
Sagittal CT showing subhepatic appendicitis (red arrow) with associated hepatic abscess in segment VI (yellow arrow).

A 56-year-old male patient was admitted to the hospital on February 28, 2025, with the chief complaint of “right abdominal pain accompanied by fever for 2 weeks.” Two weeks prior, the patient developed right abdominal pain without any obvious trigger, which persisted and was unrelieved, accompanied by fever, with a maximum temperature of 39°C. He did not experience chills, had cloudy urine, but did not report urinary frequency, urgency, dysuria, nausea, vomiting, or diarrhea. The patient has no history of night sweats, no recent weight loss, and no previous symptoms of chronic abdominal pain. The patient had a body mass index (BMI) of 29.4, with no history of smoking or alcohol consumption. He had no significant past medical history and reported no known familial or hereditary disorders. The patient sought medical attention at a local hospital, where a urinary system CT(Computed Tomography) scan revealed bilateral kidney stones. He was diagnosed with “bilateral kidney stones complicated by urinary tract infection” and was treated with cefotaxime for 10 days, but his pain did not show significant improvement. He later presented to our emergency department, where a non-contrast abdominal and pelvic CT scan (**Figures 1**, **2**) revealed acute appendicitis (subhepatic appendix) and a hepatic abscess in segment VI measuring approximately 22 mm × 29 mm. He was admitted to our department for further diagnosis and treatment.

Abdominal examination revealed tenderness in the right abdomen with no rebound tenderness, but suspicious muscle rigidity. White blood cell count: 10.19 x 10^9/L.Neutrophil count: 7.37 x 10^9/L.C-reactive protein (CRP): 53.7 mg/L. Admission diagnosis were Acute appendicitis and Hepatic abscess (segment VI).

The patient underwent laparoscopic appendectomy and drainage of the hepatic abscess under general anesthesia on the same day. Intraoperatively, the appendix was found to be located in a subhepatic position, with a swollen tip surrounded by adhesions. A hepatic abscess in segment VI was identified and was considered to be secondary to inflammatory irritation from the inflamed appendiceal tip. The abscess was successfully drained, and two abdominal drainage tubes were placed.

Postoperatively, the patient received continued fluid resuscitation and anti-inflammatory treatment. His recovery was satisfactory, with the patient passing gas on the second postoperative day. The abdominal drainage tubes were removed on the 6th and 8th days post-surgery, respectively. Infection markers gradually decreased, and the patient was discharged on the 9th postoperative day.

**Figure 3:**
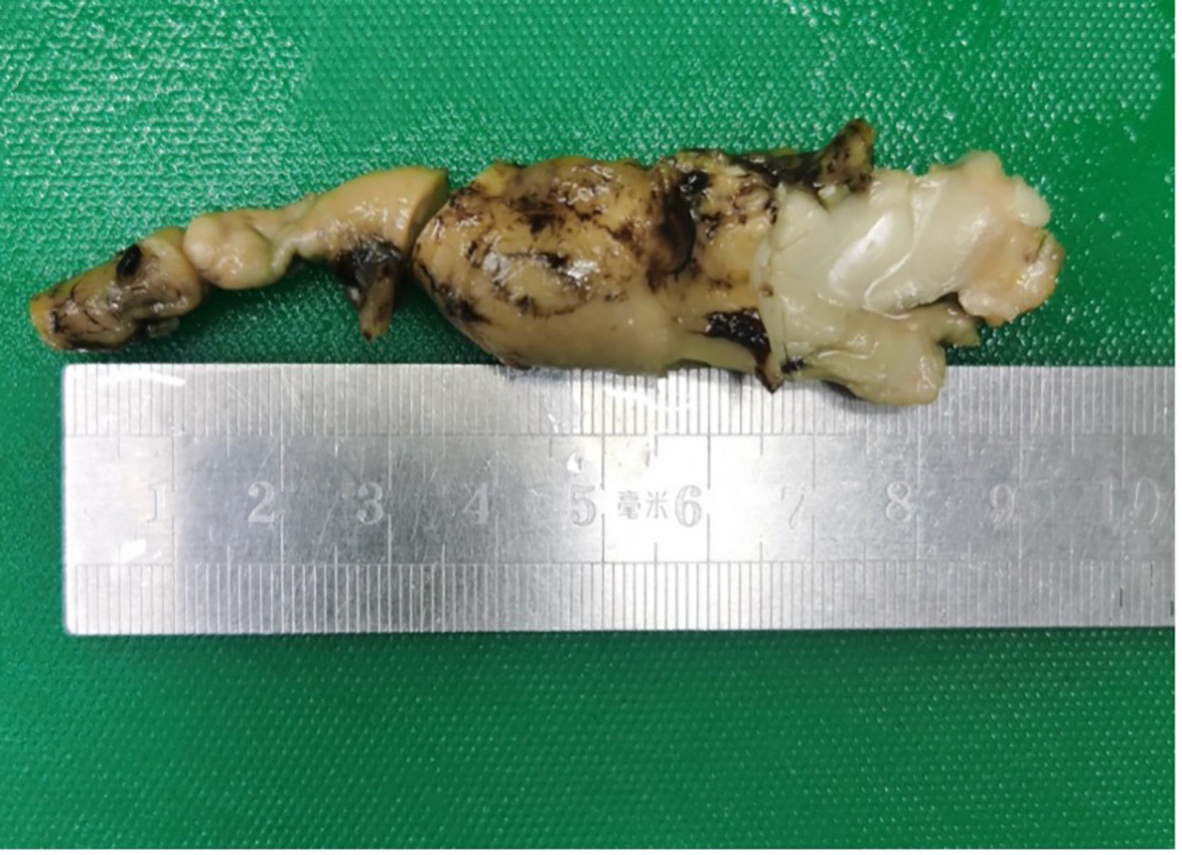
Gross specimen of the appendix.

**Figure 4:**
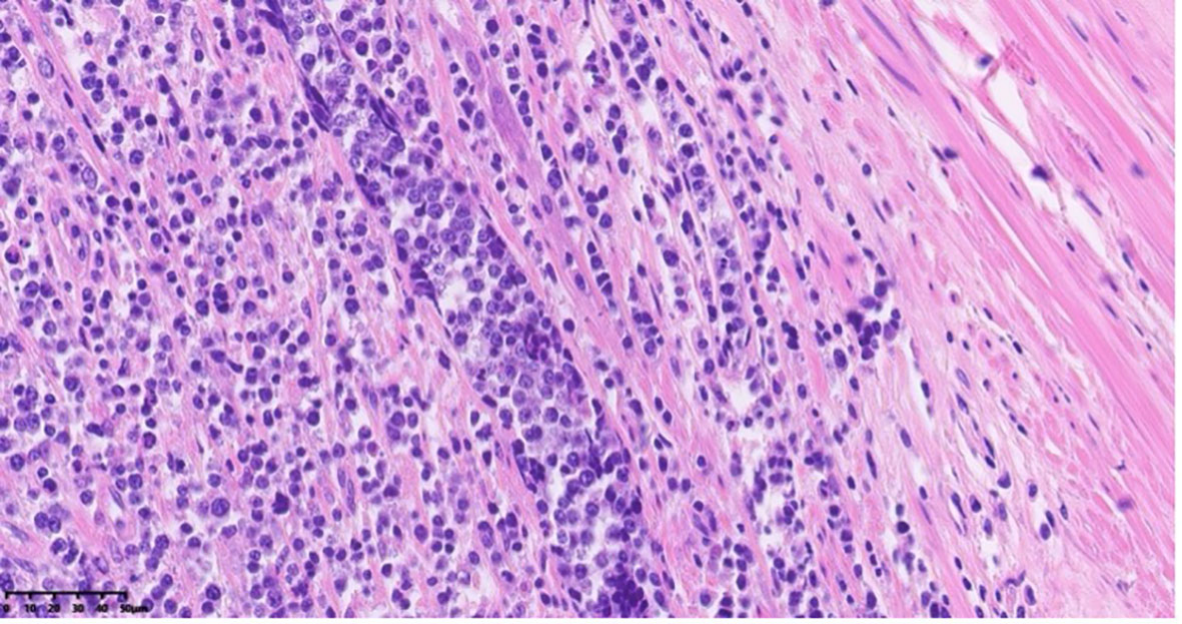
Histopathological section of appendiceal tissue with H&E staining under 40x objective lens magnification using light microscopy.

The appendix specimen measured 9 cm in length and 0.8 to 1.9 cm in diameter. (**Figure 3**) The serosal surface was mildly congested with slight fibrinous adhesions. The wall structure remained intact, and the blind end was dilated and thickened, measuring 5 cm in length and 1.9 cm in diameter. The cut surface was porcelain white. Histopathological examination revealed lymphoma involving the tip and part of the body of the appendix. The surgical margins were negative, with no evidence of tumor infiltration at the resection edges. Further analysis (**Figure 4**) demonstrated diffuse B-cell lymphocytic infiltration throughout the full thickness of the appendiceal wall, consistent with a diagnosis of non-Hodgkin lymphoma, most likely high-grade B-cell lymphoma.

**Figure 5:**
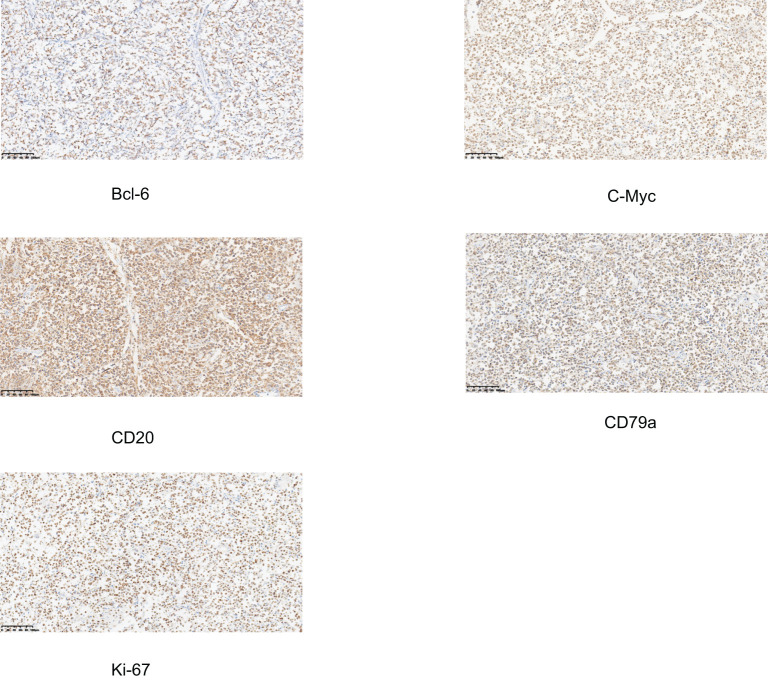
Immunohistochemical staining of the appendiceal specimen. All markers show positive expression in tumor cells, supporting the diagnosis of B-cell lymphoma. The images demonstrate positive staining for Bcl-6, C-Myc, CD20, CD79a, and Ki-67.

Immunohistochemistry (**Figure 5**) showed c-Myc (+), Bcl-6 (+), and involvement of the mesenteric fat surrounding the appendix. The immunohistochemical profile was as follows: CD3 (-), CD20 (+), CD21 (-), CD79a (+), CD38 (-), CD138 (-), Bcl-6 (+), Bcl-2 (-), Kappa (-), Lambda (-), Ki-67 (90-95%), CD10 (-), MUM1 (-), Cyclin D1 (-), CD5 (-), CD30 (-), c-Myc (+), TdT (-), Granzyme B (-), CD56 (-), CD34 (-).

Postoperatively, the patient underwent chest and head CT scans, with no abnormalities reported. According to the Ann Arbor staging system, the patient was classified as Stage I postoperatively. One month after surgery, adjuvant chemotherapy was administered using the R-CHOP regimen, which includes rituximab, cyclophosphamide, doxorubicin, vincristine, and prednisone.

## Discussion

Subhepatic appendicitis is an uncommon but clinically significant condition that occurs when the appendix is located beneath the liver, often as a result of anatomical variations such as malrotation or abnormal positioning during embryogenesis (1). The first clinical case was documented by King in 1955 (3). This anatomic abnormality predisposes the appendix to inflammation and infection that can extend to adjacent structures, particularly the liver. The liver’s close proximity to the appendix in these cases facilitates the spread of infection, which can lead to the formation of a hepatic abscess, typically in segment VI of the liver, where the inflamed appendix is often situated (4).

Clinically, patients with subhepatic appendicitis may present with right upper quadrant pain, fever, and signs of systemic inflammatory response. These symptoms often mimic other abdominal pathologies, such as cholecystitis or liver abscess, which complicates the diagnosis. Inflammatory markers like leukocytosis and elevated C-reactive protein (CRP) levels are commonly observed, but they are non-specific and should prompt clinicians to consider imaging as the next step in diagnosis.

Imaging modalities, particularly abdominal contrast-enhanced computed tomography (CT), play a crucial role in diagnosing subhepatic appendicitis and its complications (5). CT imaging reveals not only the appendiceal inflammation but also any associated hepatic abscesses, which typically appear as hypo- or anechoic areas with or without gas formation. The diagnosis of hepatic abscess secondary to appendicitis is confirmed when pus or fluid collection is noted in proximity to the inflamed appendix.

Management of subhepatic appendicitis complicated by hepatic abscess requires a multidisciplinary approach. Surgical intervention, typically in the form of laparoscopic appendectomy, is the cornerstone of treatment. The removal of the appendix not only resolves the source of infection but also minimizes the risk of recurrence. Simultaneously, hepatic abscesses should be drained, either percutaneously or surgically, to facilitate resolution of the infection. Postoperative care involves administration of broad-spectrum antibiotics, with adjustment based on culture and sensitivity results. In severe cases, additional interventions such as peritoneal drainage or prolonged antibiotic therapy may be required.

The prognosis of patients with subhepatic appendicitis complicated by hepatic abscess is generally favorable if timely surgical intervention and appropriate drainage are performed. However, delayed treatment can lead to severe complications such as sepsis, hepatic failure, and multi-organ dysfunction.

Primary diffuse large B-cell lymphoma (DLBCL) is the most common and aggressive subtype of non-Hodgkin lymphoma (NHL), characterized by the monoclonal proliferation of large, malignant B-cells. Although DLBCL typically arises from lymphoid tissues such as lymph nodes or the spleen, primary extranodal DLBCL can involve almost any organ, including the gastrointestinal tract, central nervous system, and even the appendix. Primary DLBCL of the appendix is an exceedingly rare occurrence (6), often presenting as a rapidly growing mass and causing nonspecific symptoms such as abdominal pain, fever, weight loss, and gastrointestinal discomfort.

Histopathologically, DLBCL is characterized by large, pleomorphic cells with prominent nucleoli and a high mitotic rate. These cells infiltrate the tissue in a diffuse pattern, and the tumor may involve the full thickness of the organ wall. In primary appendiceal DLBCL, the appendix becomes significantly enlarged and may exhibit signs of perforation, with the tumor potentially extending to adjacent tissues such as mesenteric fat or the peritoneal cavity.

Immunohistochemistry is crucial for the definitive diagnosis of DLBCL and differentiates it from other lymphoma subtypes and malignancies. The hallmark markers for DLBCL are CD20 and CD79a, which are positive in most cases. Additionally, Bcl-6 is commonly expressed in the majority of high-grade DLBCLs, and c-Myc overexpression, observed in about 30% of cases, is associated with a more aggressive form of the disease (7). Other immunohistochemical markers typically include Bcl-2 (negative or low expression), CD3 (negative), CD10 (negative), and Ki-67 (positive, often >90%), which reflects a high proliferative index. The use of molecular profiling, including gene rearrangements and mutations in the MYC, BCL2, and BCL6 genes, can help refine the diagnosis and provide prognostic information.

Diffuse Large B-Cell Lymphoma (DLBCL) requires careful differentiation from other conditions like chronic infections, metastatic carcinomas, and other lymphomas. PET-CT plays a key role in staging, evaluating treatment response, and distinguishing DLBCL from benign or inflammatory conditions by detecting metabolic activity in affected tissues (8). Fluid biomarkers, particularly circulating tumor DNA (ctDNA), are emerging as non-invasive tools for assessing minimal residual disease, genetic mutations, and treatment efficacy. ctDNA can also provide insights into clonal evolution and disease progression (9). Omics research, including genomics, transcriptomics, and proteomics, enhances our understanding of DLBCL by revealing genetic mutations, gene expression profiles, and protein dysregulation, which can inform targeted therapies and prognostic predictions (10). Integration of these approaches will lead to personalized medicine, improving diagnosis, treatment, and outcomes for DLBCL patients.

Treatment of primary DLBCL involves a combination of chemotherapy, typically the R-CHOP regimen (rituximab, cyclophosphamide, doxorubicin, vincristine, and prednisone), and in some cases, radiation therapy (11). The therapeutic approach is guided by the tumor’s stage, presence of extranodal involvement, and overall patient health (12). Surgery is rarely needed except in cases of localized disease or complications such as bowel perforation, obstruction, or bleeding.

The prognosis for patients with primary DLBCL is influenced by several factors, including the International Prognostic Index (IPI), which incorporates factors such as age, performance status, extranodal involvement, and serum lactate dehydrogenase (LDH) levels (13). In general, DLBCL is highly responsive to initial chemotherapy, with most patients achieving complete remission (14). However, the prognosis can vary significantly, with patients harboring high-risk features or advanced-stage disease having a more guarded outlook. Relapse is common in patients with high-grade DLBCL, necessitating the use of second-line chemotherapy regimens or stem cell transplantation in refractory cases (15).

## Conclusions

This case highlights the diagnostic challenges posed by subhepatic appendicitis complicated by hepatic abscess, particularly when associated with rare malignancies such as primary diffuse large B-cell lymphoma. Clinicians should remain vigilant for atypical presentations, and thorough pathological examination is essential for accurate diagnosis and appropriate management.

## Data Availability

The original contributions presented in the study are included in the article/supplementary material. Further inquiries can be directed to the corresponding author/s.

## References

[1] Isreb S Holtham S. Incidental finding of an anterior sub-hepatic appendix during laparoscopic cholecystectomy. BMJ Case Rep. (2010) 2010:bcr0420102883. doi: 10.1136/bcr.04.2010.2883 PMC3029877 22778286

[2] Yousef AH Suleimanov V. Subhepatic appendicitis presenting with recurrent abdominal pain. Cureus. (2022) 14:e32939. doi: 10.7759/cureus.32939 36712760 PMC9873595

[3] King A. Subhepatic appendicitis. AMA Arch Surg. (1955) 71:265–7. doi: 10.1001/archsurg.1955.01270140113021 14397970

[4] Armstrong T Dluzewski S Yu D. Appendicitis with direct fistulation into the liver: a forgotten cause of pyogenic liver abscess. BJR Case Rep. (2020) 6:20200101. doi: 10.1259/bjrcr.20200101 33299600 PMC7709073

[5] Algın O Özmen E Özcan AS Erkekel S Karaoğlanoğlu M. Unusual manifestation of acute retrocecal appendicitis: pericholecystic fluid. Ulus Travma Acil Cerrahi Derg. (2013) 19:80–2. doi: 10.5505/tjtes.2013.74508 23588987

[6] García-Norzagaray JC Villalobos-López JA Flores-Nájera H ValleLeal JG García Torres CD. Primary lymphoma of the appendix: a case report and review of the literature. Rev Gastroenterol Mex. (2019) 84:254–7. doi: 10.1016/j.rgmxen.2018.07.003 29807767

[7] Sukswai N Lyapichev K Khoury JD Medeiros LJ. Diffuse large B-cell lymphoma variants: an update. Pathology. (2019) 52:53–67. doi: 10.1016/j.pathol.2019.08.013 31735345

[8] Rösler W Bink A Bissig M Imbach L Marques Maggio E Manz MG. CAR T-cell infusion following checkpoint inhibition can induce remission in chemorefractory post-transplant lymphoproliferative disorder of the CNS. Hemasphere. (2022) 6:e733. doi: 10.1097/HS9.0000000000000733 35747591 PMC9208876

[9] Semenkovich NP Szymanski JJ Earland N Chauhan PS Pellini B Chaudhuri AA. Genomic approaches to cancer and minimal residual disease detection using circulating tumor DNA. J Immunother Cancer. (2023) 11:e006284. doi: 10.1136/jitc-2022-006284 37349125 PMC10314661

[10] Liang XJ Song XY Wu JL Liu D Lin BY Zhou HS. Advances in multi-omics study of prognostic biomarkers of diffuse large B-cell lymphoma. Int J Biol Sci. (2022) 18:1313–27. doi: 10.7150/ijbs.67892 PMC8898353 35280688

[11] Cabanillas F Shah B. Advances in diagnosis and management of diffuse large B-cell lymphoma. Clin Lymphoma Myeloma Leukemia. (2017) 17:783–96. doi: 10.1016/j.clml.2017.10.007 29126866

[12] Abdalla MF El-Hennawy HM. Unusual presentation for pri-mary appendiceal lymphoma: a case report. Ind J Surg. (2010) 72:289–92. doi: 10.1007/s12262-010-0093-5 PMC3451848 23133274

[13] Solal-Céligny Roy P Colombat P White J Armitage JO Arranz-Saez R. Follicular lymphoma international prognostic index. Blood. (2004) 104:1258–65. doi: 10.1182/blood-2003-12-4434 15126323

[14] Gisselbrecht C Van Den Neste E. How I manage patients with relapsed/refractory diffuse large B cell lymphoma. Br J Haematol. (2018) 182:633–43. doi: 10.1111/bjh.15412 PMC6175435 29808921

[15] Chavez JC Locke FL. CAR T cell therapy for B-cell lymphomas. Best Pract Res Clin Haematol. (2018) 31:135–46. doi: 10.1016/j.beha.2018.04.001 PMC6716161 29909914

